# Blood Pressure Control and Recurrent Stroke After Intracerebral Hemorrhage in 2002 to 2018 Versus 1981 to 1986: Population-Based Study

**DOI:** 10.1161/STROKEAHA.121.034432

**Published:** 2021-07-08

**Authors:** Linxin Li, Susanna M. Zuurbier, Wilhelm Kuker, Charles P. Warlow, Peter M. Rothwell

**Affiliations:** 1 Wolfson Centre for Prevention of Stroke and Dementia, Nuffield Department of Clinical Neuroscience, University of Oxford, United Kingdom (L.L., S.M.Z., W.K., C.P.W., P.M.R.).; 2 Department of Clinical Neurosciences, University of Edinburgh, United Kingdom (C.P.W.).

**Keywords:** blood pressure, cerebral hemorrhage, ischemic stroke, risk, survivors

## Abstract

The PROGRESS trial (Perindopril Protection Against Recurrent Stroke Study) conducted in the early 1990s showed that blood pressure (BP) lowering therapy reduced the risks of recurrent stroke by about 50% after spontaneous intracerebral hemorrhage (ICH). However, the ICH subgroup was a minority, and trial cohorts are invariably selective. Therefore, it is unclear whether the impact of BP control on risk of recurrent stroke in ICH observed in PROGRESS would be as great in real-world practice.

**Methods:**

We compared BP control (mean BP during study follow-up) and risks of recurrent stroke after first-ever primary ICH in 2 colocated population-based studies before and after the PROGRESS trial (1995–2001) in Oxfordshire: Oxfordshire Community Stroke Project (OCSP; 1981–1986) and OXVASC (Oxford Vascular Study; 2002–2018).

**Results:**

Two hundred seventy-seven patients (753 patient-years of follow-up) with first-ever primary ICH were ascertained in OXVASC and OCSP. Baseline systolic BP was comparable between the 2 studies (mean/SD=183.8/36.5 in OXVASC versus 178.1/38.2 in OCSP, *P*=0.30) but among one hundred thirty-seven 90-day survivors, mean BP during follow-up was substantially lower in OXVASC versus OCSP (135.2/16.4 versus 157.3/17.8, *P*<0.0001). Risks of recurrent stroke (per 100 patient-years) decreased from 10.3 (95% CI, 4.7–19.5) in OCSP to 3.1 (1.8–4.8) in OXVASC (*P*=0.006), predominantly driven by a reduction at younger ages (5-year risk at age <75 years: 35.4% versus 6.9%, *P*=0.001; hazard ratio, 0.14 [0.04–0.54]).

**Conclusions:**

Risks of recurrent stroke after primary ICH have fallen significantly in Oxfordshire over the past 4 decades, coinciding with substantial improvements in BP control during follow-up.

## Abstract 2

Supplemental Digital Content is available in the text.

The PROGRESS trial (Perindopril Protection Against Recurrent Stroke Study), conducted in the early 1990s, showed that effective blood pressure (BP) lowering with perindopril and indapamide reduced the risks of recurrent stroke in patients with recent cerebrovascular events,^1^ with the strongest effect (49% risk reduction) in those with spontaneous intracerebral hemorrhage (ICH).^2^ However, the ICH subgroup was small and will have been somewhat selected for inclusion in the trial. Therefore, it is unclear whether the impact of BP control on the risk of recurrent stroke in ICH observed in PROGRESS would be as great in real-world practice.

In contrast to ischemic stroke, secondary prevention treatment options after ICH other than BP lowering have changed little since the 1980s. Although diagnosis and treatment of hypertension in primary prevention have improved since the 1980s,^3^ and incidence of hypertension-related ICH has fallen,^4^ there are a few published data on time trends in risk of recurrent stroke after ICH in secondary prevention. Given the strong association between BP and ICH in secondary prevention,^5^ we hypothesized that the long-term risks of recurrent stroke after ICH might have also improved alongside improvement in BP control over time consequent upon the findings from PROGRESS. Using data from 2 population-based studies in Oxfordshire, United Kingdom, that were conducted before and after the PROGRESS trial (Oxfordshire Community Stroke Project [OCSP]; 1981–1986; OXVASC [Oxford Vascular Study] 2002–2018), we, therefore, aimed to determine the time trends of BP control and long-term risks of recurrent stroke after a first-ever primary ICH.

## Methods

Requests for access to data from the Oxford Vascular Study will be considered by the corresponding author.

OXVASC is an ongoing population-based study of the incidence and outcome of all acute vascular events in a population of 92 728 individuals, registered with about 100 general practitioners in 9 general practices in Oxfordshire, United Kingdom. The multiple overlapping methods used to achieve near-complete ascertainment of all individuals with stroke have been reported previously.^6^ Briefly, these included (1) a daily, rapid-access transient ischemic attack/stroke clinic to which participating general practitioners and the local emergency department team referred individuals with suspected transient ischemic attack or minor stroke; (2) daily searches of admissions to medical, stroke, neurology, and other relevant wards; (3) daily searches of the local emergency department attendance register; (4) daily searches of in-hospital death records via the bereavement office; (5) monthly searches of all death certificates and coroner’s reports for out-of-hospital deaths; (6) monthly searches of general practitioner diagnostic coding and hospital discharge codes; and (7) monthly searches of brain and vascular imaging referrals.

Patients with suspected stroke were seen by study physicians as soon as possible after the initial presentation. Demographic data and vascular risk factors before the event were collected from face-to-face interview and cross-referenced with primary care records. If a patient died before assessment, we obtained an eyewitness account of the clinical event and reviewed any relevant records. All cases were reviewed by the senior study neurologist (P.M. Rothwell) for final adjudication. The rate of imaging, autopsy, or both was 96% in OXVASC.^4^

A computed tomography-based imaging protocol was used for patients with suspected ICH, and the study neuroradiologist (W. Kuker) reviewed all scans. Cases were also screened for underlying causes by magnetic resonance brain or by angiography, especially when the ICH occurred in those below the age of 50 years or in the absence of other risk factors.^4^ For the current analysis, only consecutive patients with first-ever primary ICH were included. Patients with ICH related to trauma, tumor, thrombolysis, or other underlying causes (ie, vascular malformation, hematological malignancy, or cerebral venous thrombosis) were excluded. Patients with isolated intraventricular hemorrhage were also not included.

All OXVASC patients had treatment of hypertension to guideline targets (<130/80 mm Hg) and perindopril arginine (±indapamide) based regimen was considered the first-line treatment.

Patients were followed-up face-to-face at 1, 6, 12, 60, and 120 months by a study nurse or physician for standardized BP measurement (2 readings taken while sitting using a digital BP monitor), functional (modified Rankin Scale) assessment and to identify any recurrent ICH or ischemic stroke supplemented by review of primary care records. Disability was defined as modified Rankin Scale between 3 and 5. Patients who had moved out of the study area were followed-up via telephone at the same time-points as face-to-face follow-up. We recorded all deaths during follow-up with the underlying causes by direct follow-up, via primary care records, and by centralized registration with Office for National Statistics. All recurrent events that occurred during follow-up would also be identified by the ongoing daily case ascertainment. If a recurrent stroke was suspected, the patient was reassessed and investigated by a study physician.

OCSP is also a high quality, population-based study of all first-ever strokes from 1981 to 1986 with an overlapping general practice population with OXVASC. The methodology of OCSP has also been published before,^5^ and the case diagnosis, assessment, and follow-up were similar to those in OXVASC. In particular, to ensure consistency of diagnosis between OCSP and OXVASC, summaries of all potential cases in the first 2 years of OXVASC were reviewed together with the principal investigator of OCSP to ensure that the application of definitions of strokes was comparable.^6^ The rate of imaging, autopsy, or both was ≈90% in OCSP.^5^

### Statistical Analysis

Baseline characteristics and follow-up BP readings were compared between OCSP versus OXVASC using the χ^2^ test for categorical variables and *t* test for continuous variables.

Estimates of risk were derived from Kaplan-Meier analyses censored at the outcome of interest, death or December 31, 1998, for OCSP and November 30, 2019, for OXVASC, whichever happened first. We compared the annual rates and 5-year risks of the following outcomes between OCSP and OXVASC: any recurrent stroke, recurrent ICH, ischemic stroke, stroke of unknown subtype, and death. Given the mean age of the ICH subgroup of PROGRESS trial was 61 years with 70% being male,^1,2^ analyses were stratified by age (<75 versus ≥75 years) and sex. Sensitivity analyses confining to patients within the same general practices between OCSP and OXVASC and to those not on antithrombotic treatment before the index ICH were performed.

Prevalence of disability at baseline, 1-, and 5-year follow-up was compared between OCSP and OXVASC using the χ^2^ test.

All analyses were done using SPSS version 25.

## Results

Overall, 277 patients with first-ever incident primary ICH cases were ascertained (n=66 from OCSP and n=211 from OXVASC). The baseline characteristics of the patients included in both studies are shown in Table 1. In OXVASC, patients tended to be older compared with OCSP, with a higher proportion of patients aged ≥75 years (n/%=133/63.0 versus 31/47.0, *P*=0.02). There was also a higher prevalence of diabetes, and premorbid use of antithrombotic treatment in OXVASC (Table 1). The prevalence of history of hypertension was, however, similar and BP assessed at baseline was also largely comparable between the 2 periods (Table 1). Although the overall prevalence of history of atrial fibrillation did not differ significantly between the 2 studies, prevalence was higher at age ≥75 years in OXVASC versus OCSP (n/%<75 years=4/11.8 versus 8/10.3, *P*=0.81; ≥75 years=1/3.4 versus 27/20.3, *P*=0.03).

**Table 1.**
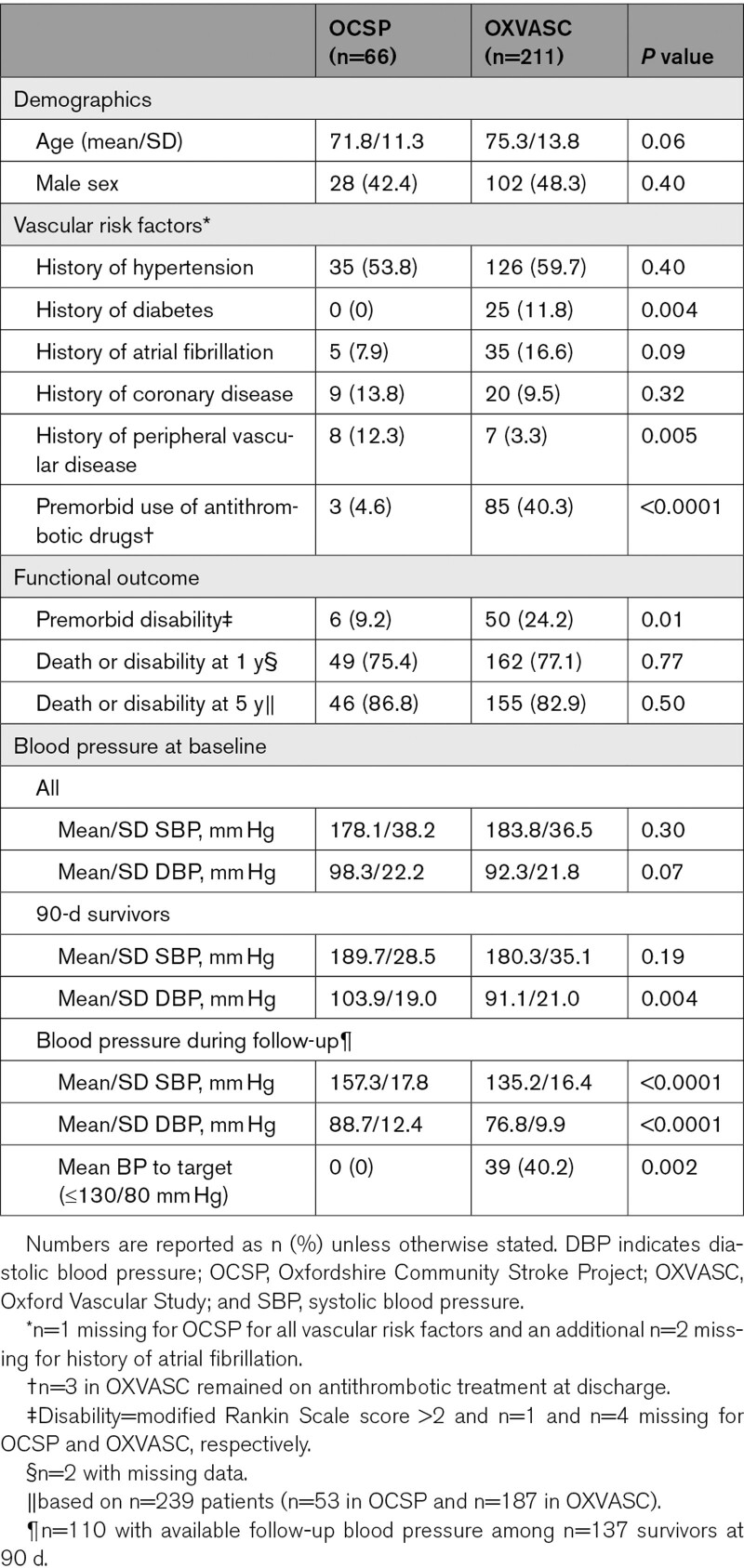
Baseline Characteristics of Patients Presenting With First-Ever Primary Intracerebral Hemorrhage in OCSP (1981–1986) and OXVASC (2002–2018)

BP control in 90-day survivors (mean level over all subsequent follow-up visits) improved significantly between OCSP and OXVASC (Table 1) and was independent of age (OCSP versus OXVASC—age<75 years: mean/SD systolic BP=159.0/20.4 versus 134.3/14.8, *P*=0.0003; age≥75 years=155.7/16.4 versus 136.1/17.8, *P*=0.005). Results were also consistent in analysis stratified by history of hypertension (Table I in the Data Supplement).

Annual rate of any recurrent stroke reduced (*P*=0.006) from 10.3 (95% CI, 4.7–19.5) in OCSP to 3.1 (1.8–4.8) per 100 patient-years in OXVASC (Table 2), with a similar trend for men and women (Table II in the Data Supplement), for those with versus without history of diagnosed hypertension (Table I in the Data Supplement), and in those not on premorbid antithrombotic treatment (8.5 [3.4–17.6] versus 2.4 [1.2–4.1] per 100 patient-years, *P*=0.01). The reduction was mainly accounted for by a lower risk after 90 days (risks at 90 days=5.7% versus 3.3% in OXVASC, *P*=0.51; post 90 days=27.2% versus 10.4% in OXVASC, *P*=0.004) and was predominantly driven by patients at younger ages (5-year risk at age <75 years=35.4% versus 6.9% in OXVASC, *P*=0.001; age≥75 years=16.3% versus 20.1%, *P*=0.59; Figure). Similar trends were found for recurrent ICH, ischemic stroke, and for recurrent strokes of unknown subtypes (Table 2 and Figure I in the Data Supplement). Results were consistent in analyses confined to patients registered within the same general practices in the 2 studies (Table 2 and Figures I and II in the Data Supplement), and the reduction in recurrent stroke was most prominent between OCSP and the first 5 years of OXVASC, which corresponded to the change of BP at follow-up (Figure III in the Data Supplement). There was no evidence of a difference in follow-up mean BP between patients with and without recurrent stroke in OXVASC (mean/SD with recurrence versus without: systolic BP 136.5/16.1 versus 135.0/16.5, *P*=0.75; diastolic BP 78.1/11.8 versus 76.5/9.6, *P*=0.58).

**Table 2.**
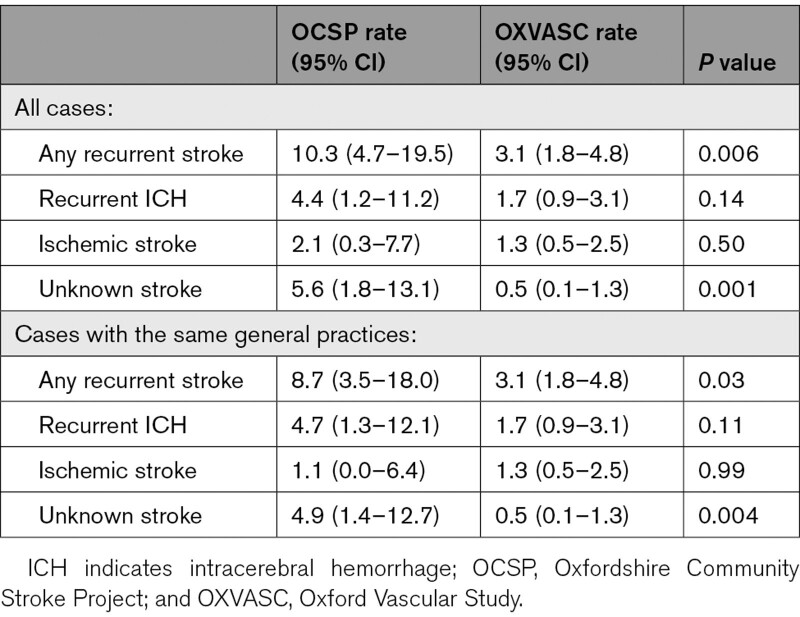
Annual Rates (per 100 Patient-Years) of Recurrent Stroke, Recurrent ICH, Ischemic Stroke, Unknown Stroke, and Death in Patients With First-Ever Primary ICH in OCSP (1981–1986) Versus OXVASC (2002–2018)

**Figure.**
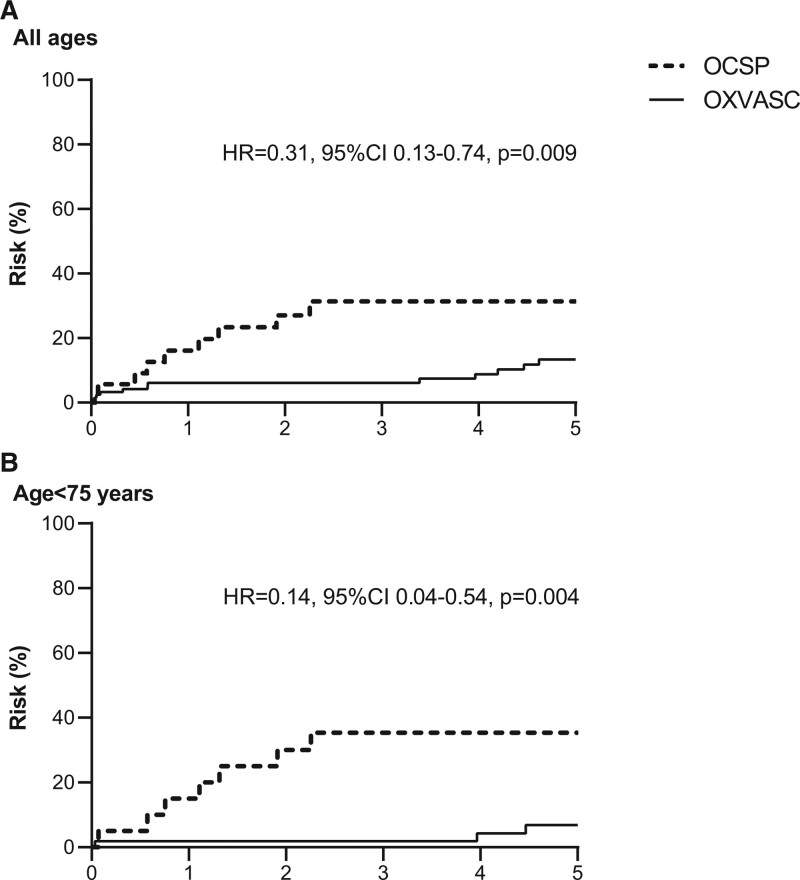
**Risks of recurrent stroke in Oxfordshire Community Stroke Project (OCSP; 1981–1986) and OXVASC (Oxford Vascular Study; 2002–2018) in patients with first-ever primary intracerebral hemorrhage. A**, All age groups; (**B**) those aged <75 years only.

We also compared the outcomes of patient with incident primary ICH in OCSP versus OXVASC. At baseline, the frequency of premorbid disability was higher in OXVASC than in OCSP (odds ratio, 3.1 [95% CI, 1.3–7.7], *P*=0.01; Table 1), which was attenuated after adjustment for age (adjusted odds ratio, 2.4 [0.9–6.1], *P*=0.07). There were also substantial risks of death at 90 days, 1 year, and 5 years (Figure II in the Data Supplement), which did not change over time (death—OCSP versus OXVASC: at 90 days=37/56.1% versus 101/48.3%, *P*=0.23; at 1 year=41/63.1% versus 116/55.0%, *P*=0.24; at 5 years=44/69.4% versus 133/64.1%, *P*=0.29). Death/disability at 1- or 5-year follow-up was also unchanged over time (Table 1). The results were largely consistent in analyses stratified by age and sex (Table III in the Data Supplement), using modified Rankin Scale score >3 as a cutoff for disability (Table IV in the Data Supplement), and in analyses confined to patients registered within the same general practices in both studies (Figure II) and for those who survived at 5 years with no disability at baseline (n/%—OCSP versus OXVASC=2/22.2% versus 16/33.3%, *P*=0.51).

## Discussion

Using data from 2 population-based studies with long-term follow-up, we showed that there was significant reduction in the risks of recurrent stroke after incident primary ICH in the past 4 decades in Oxfordshire, United Kingdom, which coincided with substantial improvement in BP control. However, there has been no change in risk of death or disability at 1 or 5 years after primary ICH.

Our findings that the reduction of recurrent stroke after ICH coincided with substantial improvement in BP control during follow-up is consistent with the findings of the PROGRESS trial. PROGRESS reported that antihypertensive treatment reduced risks of recurrent ICH by 49% with a reduction of 11/4 mm Hg in BP,^2^ which was comparable to what we found in patients aged <75 years who had a similar mean age to those in the trial. Our results also highlight the potential importance of more intensive management of BP after ICH. Several clinical trials are currently underway to answer this question (TRIDENT [Triple Therapy Prevention of Recurrent Intracerebral Disease Events Trial], URL: https://www.clinicaltrials.gov; Unique identifier: NCT02699645 and PROHIBIT-ICH [Prevention of Hypertensive Injury to the Brain by Intensive Treatment in IntraCerebral Haemorrhage], URL: https://www.clinicaltrials.gov; Unique identifier: NCT03863665^7^).

Despite reduction of risks of recurrent stroke in the longer term, the high early risk of recurrence remained unchanged in our study. Several other studies have also showed that risks of recurrence were particularly high in the first few days or months after ICH,^8,9^ highlighting the need for more effective treatment early after primary ICH. Randomized trials have showed that stroke unit care and early and stable acute BP lowering could be beneficial,^10,11^ while there are still uncertainties about the role of minimally invasive surgery or hemostatic drugs.^12,13^ Given that early recurrence is likely to be related to the underlying cause of ICH, new treatments targeting secondary injuries and the other underlying causes of ICH, such as cerebral amyloid angiopathy, are perhaps also needed.

Although our numbers were small in analyses stratified by age, the finding that the reduction of risks of recurrent stroke in OXVASC compared with OCSP was less apparent at older ages might be plausible. At baseline older patients were more likely to have known atrial fibrillation in OXVASC than in OCSP and were also more likely to be on antiplatelet treatment before the ICH, and most patients had their antithrombotic treatment stopped after the primary ICH. We also found that long-term risks of death or disability did not change over time, which was in line with previous studies from the United States,^14^ and Australia,^15^ although there was a trend towards improved outcomes at age younger than 75 years, consistent with a large administrative coding based study in the Netherlands.^16^ One previous study reported improved 10-year survival between 1999 and 2007 in patients hospitalized for ICH in Finland, but the difference was accounted for by patients in residential care, suggesting largely unchanged functional outcome.^17^

The strength of our study is its population-based design with near-complete ascertainment for both studies and for both the index and the recurrent events. However, the study has limitations. First, the overall number of ICH was still small, limiting our statistical power to detect time trends for subgroup analyses stratified by age, sex, and for different stroke subtypes. Second, there was limited information on hematoma location in OCSP. Therefore, we were not able to determine if the observed reduction in the risks of recurrent stroke after primary ICH over time differed by hematoma location. Third, we did not have details of the severity of the events at baseline in OCSP; hence, we could not test if any change of ICH severity contributed to the time trends of the observed long-term outcome between the 2 studies. However, short-term case-fatality was also unchanged between OCSP and OXVASC. Fourth, mortality and morbidity after primary ICH are partly explained by multiple other factors,^18^ such as initial hematoma volume and acute hematoma expansion, which were not measured. Fifth, statin use, especially in primary prevention, has also increased significantly between OCSP and OXVASC. However, initiation of statin is not recommended after ICH and only a few patients remained on premorbid statin after the ICH event. Sixth, all but 3 patients had antithrombotic treatment stopped during follow-up so we were not able to test if there was any association between use of antithrombotic drug and risk of recurrent stroke. Nevertheless, the observed reduction in recurrent stroke in our study was unlikely explained by the use of antithrombotic treatment. Recent and ongoing randomized trials addressing the impact of continuing versus stopping prior antithrombotic treatment might shed light on how best to reduce the risks of recurrent stroke, especially risks of recurrent ischemic stroke in these patient groups.^19^ Seventh, availability of different types of antihypertensive drugs as well as dosing recommendation evolved substantially between OCSP and OXVASC. Unfortunately, we did not have detailed information on dosing and types of antihypertensive drugs in OCSP for a fair comparison between the 2 cohorts. Finally, our results are based on a predominantly White population and might not be generalizable to other countries, especially Asian populations where the pattern of recurrence has been suggested to differ.^20^

## CONCLUSIONS

In conclusion, we showed that risks of recurrent stroke after primary ICH have fallen significantly in Oxfordshire over the past 4 decades, which coincided with substantial improvement in BP control, providing evidence of impact of the PROGRESS trial at the population level, and also supporting the need for ongoing BP lowering trials in secondary prevention of ICH. However, there has been no improvement in case-fatality, highlighting the need for improvements in acute treatment and in primary prevention if we are to reduce the overall burden of ICH.

## Acknowledgments

We are grateful to all the staff in the general practices that collaborated in the OXVASC (Oxford Vascular Study): Abingdon Surgery, Stert St, Abingdon; Malthouse Surgery, Abingdon; Marcham Road Family Health Centre, Abingdon; The Health Centre, Berinsfield; Key Medical Practice; Kidlington; 19 Beaumont St, Oxford; East Oxford Health Centre, Oxford; Church Street Practice, Wantage. We also acknowledge the use of the facilities of the Acute Vascular Imaging Centre (Oxford, United Kingdom). This work uses data provided by patients and collected by the National Health Service (NHS) as part of their care and support and would not have been possible without access to this data. The National Institute for Health Research (NIHR) recognizes and values the role of patient data, securely accessed and stored, both in underpinning and leading to improvements in research and care. Standard Protocol Approvals, Registrations, and Patient Consents: Written informed consent or assent from relatives was obtained in all participants. OXVASC (Oxford Vascular Study) was approved by the local research ethics committee (OREC A: 05/Q1604/70). Dr Li collected data, did the statistical analysis and interpretation, wrote and revised the article. Dr Zuurbier and Dr Kuker collected the data and revised the article. C.P. Warlow was the principle investigator of OCSP and revised the article. P.M. Rothwell conceived and designed the overall study, provided study supervision and funding, acquired, analyzed, and interpreted data, and wrote and revised the article.

## Sources of Funding

The OXVASC (Oxford Vascular Study) is funded by the National Institute for Health Research (NIHR) Oxford Biomedical Research Centre (IS-BRC-1215-20008), Wellcome Trust 104040/Z/14/Z, Wolfson Foundation and the European Union’s Horizon 2020 programme (grant 666881, SVDs@target). Dr Li is in receipt of a fellowship award from the Medical Research Foundation (MRF). For the purpose of Open Access, the author has applied a CC BY public copyright licence to any Author Accepted Manuscript version arising from this submission. The views expressed are those of the author(s) and not necessarily those of the NHS, the NIHR, or the Department of Health.

## Disclosures

P.M. Rothwell reports personal fees from Bayer and personal fees from BMS outside the submitted work. The other authors report no conflicts.

## Supplemental Materials

Online Tables I–IV

Online Figures I–III

## Supplementary Material


