# Supplementary material for: Blood Pressure Control and Recurrent Stroke After Intracerebral Hemorrhage in 2002 to 2018 Versus 1981 to 1986: Population-Based Study
Source: Stroke. 2021 Jul 8;52(10):3243–8. doi: 10.1161/STROKEAHA.121.034432 (PMC8478103; doi:10.1161/STROKEAHA.121.034432)
Supplement: Supplementary file 1 [file str-52-3243-s001.pdf]

Blood pressure control and recurrent stroke after intracerebral hemorrhage in 2002-18 vs 1981-86: population-based study

Linxin Li, Susanna M. Zuurbier, Wilhelm Kuker, Charles P Warlow, Peter M. Rothwell

Supplemental Materials

Table I Annual rates of recurrent stroke and mean blood pressure during follow-up in patients with first-ever primary intracerebral haemorrhage in OCSP (1981-1986) vs. OXVASC (2002-2018)

	**OCSP**	**OXVASC**	p
**Previous hypertension**			
Risks of recurrent stroke (n/per 100 patient years)	4/8.6	10 (3.4)	0.14
Mean/SD SBP during follow-up (mmHg)*	158.3/24.3	136.9/16.0	0.003
Mean/SD DBP during follow-up (mmHg)*	94.0/10.5	76.8/10.2	0.0001
**No previous hypertension**			
Risks of recurrent stroke (n/per 100 patient years)	4/10.1	9/2.7	0.05
Mean/SD SBP during follow-up (mmHg)*	154.4/10.3	132.3/15.4	0.001
Mean/SD DBP during follow-up (mmHg)*	84.7/13.6	76.7/9.2	0.06

*in 90-day survivors.

Table II Cumulative 5-year risks of recurrent stroke and death in OCSP (1981-1986) and OXVASC (2002-2018) in patients with first-ever primary intracerebral haemorrhage stratified by sex

	**OCSP** (n/cumulative risk)	**OXVASC** (n/cumulative risk)	p	Age/sex-adjusted p
**Recurrent stroke**				
Male	4/25.0%	5/12.2%	0.09	0.04
Female	5/38.7%	7/14.3%	0.02	0.03
**Death**				
Male	17/64.6%	65/64.9%	0.65	0.44
Female	27/73.0%	68/63.5%	0.04	0.03

Table III Prevalence of disability at baseline, 1-year and 5-year follow-up in patients with first-ever primary ICH in OCSP (1981-1986) vs. OXVASC (2002-2018)

	OCSP (n%)	OXVASC (n/%)	p	Age/sex-adjusted p
**Baseline disability**				
Stratified by sex:				
Male	1/3.6	18/17.8	0.06	0.33
Female	5/13.5	32/30.2	0.05	0.07
Stratified by age:				
<75 years	2/5.9	8/10.4	0.45	0.47
≥75 years	4/12.9	42/32.3	0.03	0.02
**Death or disability at 1 year**				
Stratified by sex:				
Male	17/60.7	74/73.3	0.20	1.00
Female	32/86.5	88/80.7	0.43	0.55
Stratified by age:				
<75 years	20/58.8	45/58.4	0.97	0.85
≥75 years	29/93.5	117/88.0	0.37	0.45
**Death or disability at 5 year**				
Stratified by sex:				
Male	17/81.0	70/78.7	0.82	0.33
Female	29/90.6	85/86.7	0.56	0.93
Stratified by age:				
<75 years	21/77.8	44/63.8	0.19	0.17
≥75 years	25/96.2	111/94.1	0.67	0.86

Table IV Prevalence of disability at baseline, 1-year and 5-year follow-up in patients with first-ever primary ICH in OCSP (1981-1986) vs. OXVASC (2002-2018) using modified Rankin Scale >3 as a cut-off for disability

	**OCSP** (n%)	**OXVASC** (n/%)	p
Premorbid disability	1 (1.5%)	16 (7.7%)	0.07
Death or disability at 1 year	44 (67.7%)	144 (68.6%)	0.89
Death or disability at 5 year	45 (84.9%)	146 (78.1%)	0.28

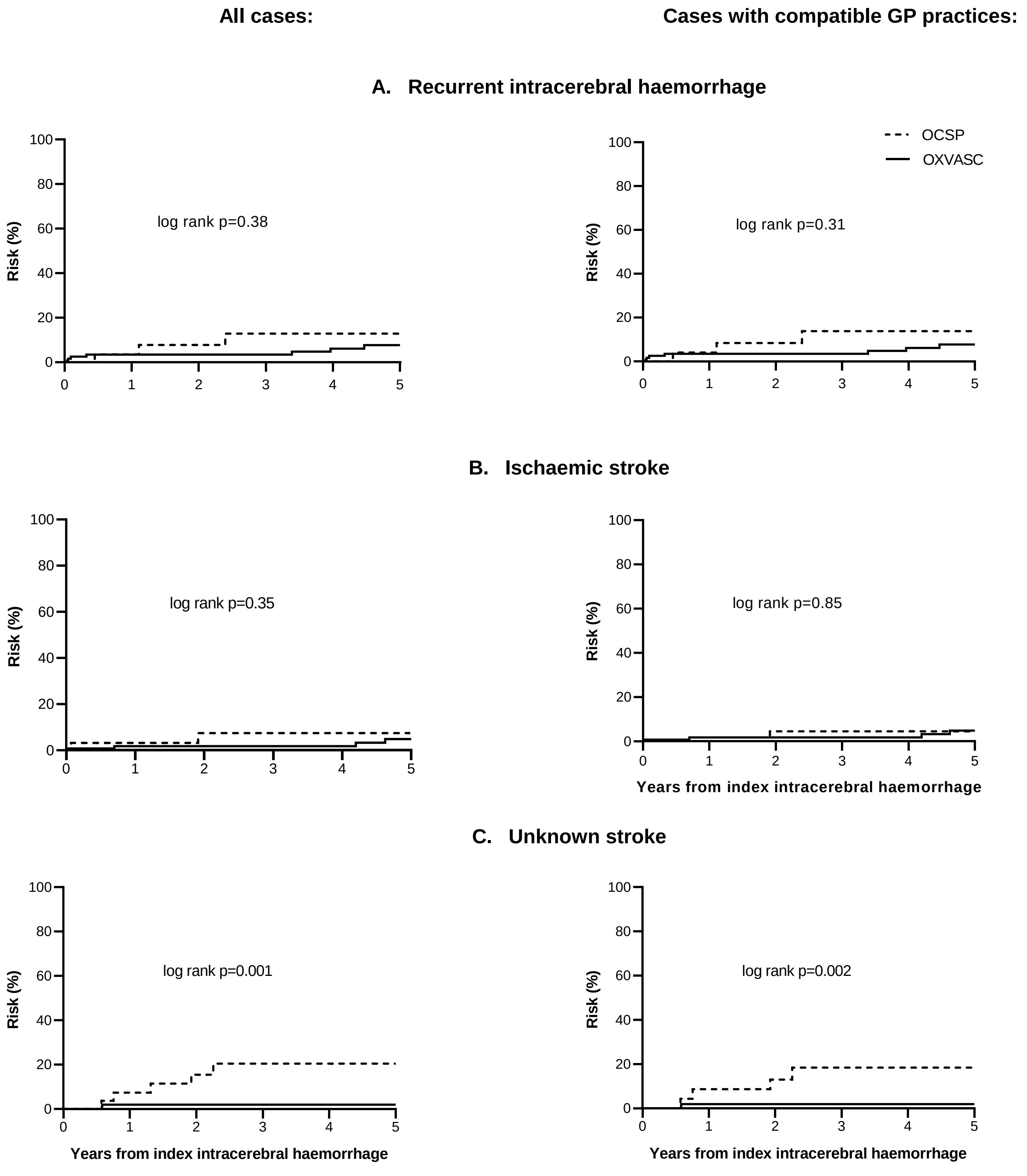

Figure I Risks of recurrent intracerebral haemorrhage and ischaemic stroke in OCSP (1981-1986) and OXVASC (2002-2018) in patients with first-ever primary intracerebral haemorrhage

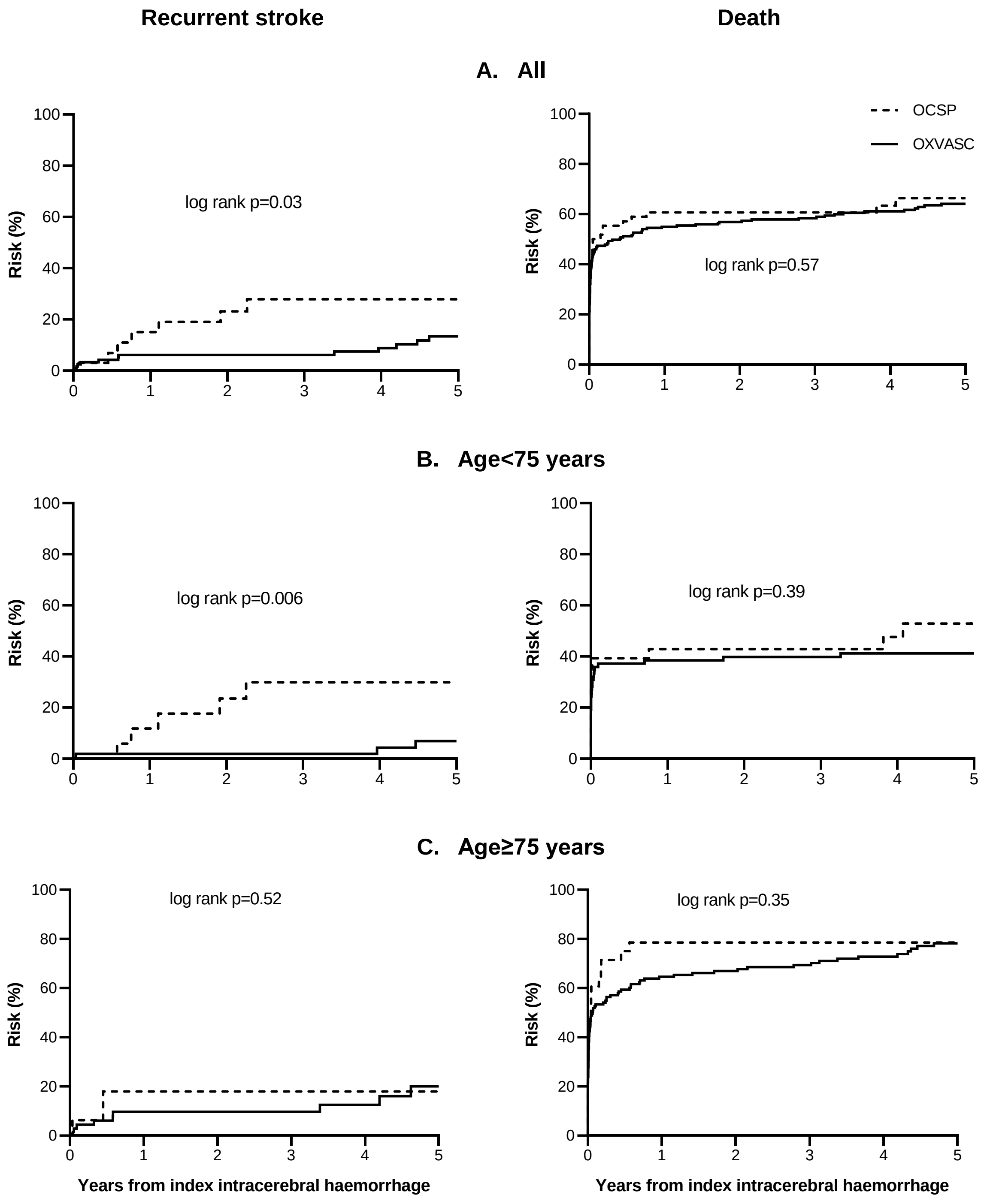

Figure II Risks of recurrent stroke and death in OCSP (1981-1986) and OXVASC (2002-2018) in patients with first-ever primary intracerebral haemorrhage including only cases within the same GP practices in both studies

Left panel for recurrent stroke for all patients (A), those aged <75 years (B) and those aged ≥75 years (C)
Right panel for death for all patients (A), those aged <75 years (B) and those aged ≥75 years (C)

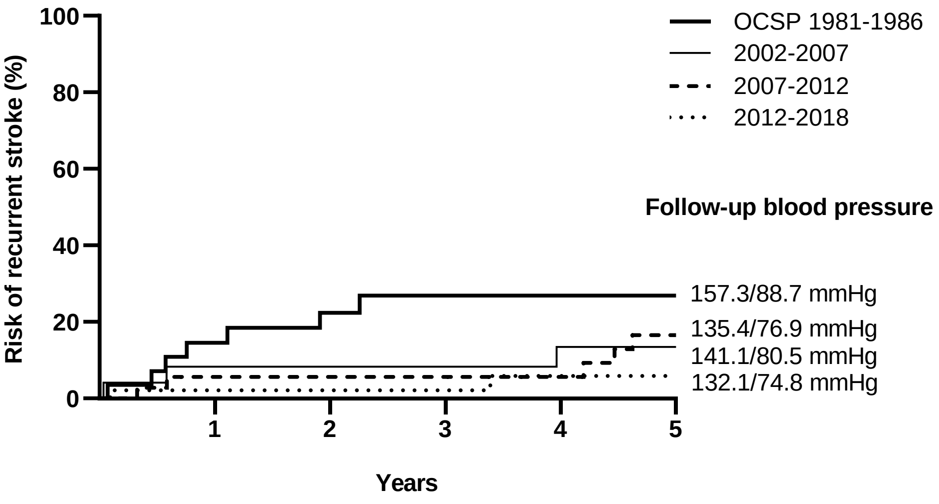

Figure III Risks of recurrent stroke and blood pressure during follow-up in OCSP (1981-1986) and different time periods within OXVASC (2002-2018) in patients who survived the first 90 days after first-ever primary intracerebral haemorrhage
