# Supplementary figures and images for: Blood Pressure Control and Recurrent Stroke After Intracerebral Hemorrhage in 2002 to 2018 Versus 1981 to 1986: Population-Based Study
Source: Stroke. 2021 Jul 8;52(10):3243–8. doi: 10.1161/STROKEAHA.121.034432 (PMC8478103; doi:10.1161/STROKEAHA.121.034432)

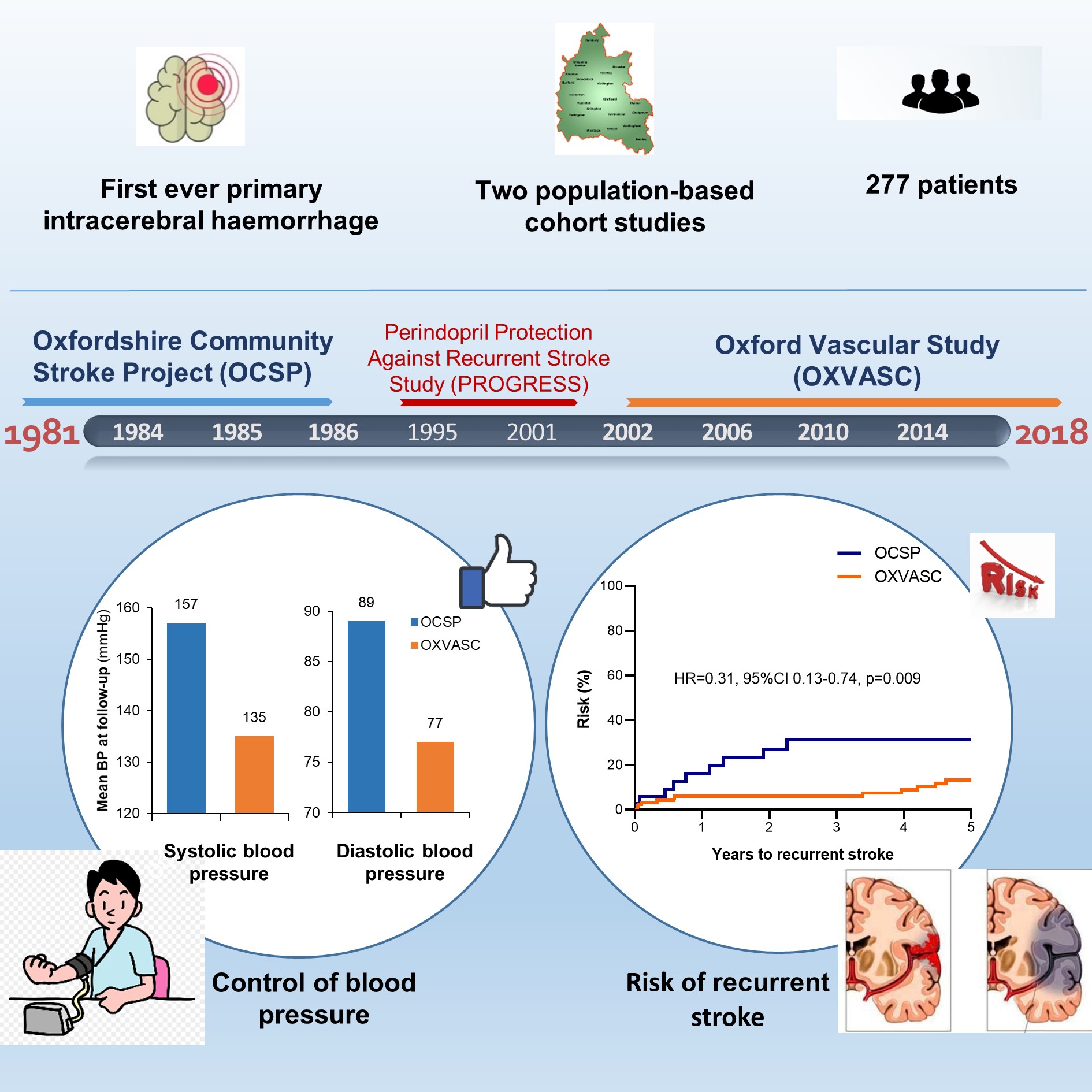

Supplement: Supplementary file 2 [file str-52-3243-s002.jpg]
